# Comparison of the effects of neostigmine and sugammadex on postoperative residual curarization and postoperative pulmonary complications by means of diaphragm and lung ultrasonography: a study protocol for prospective double-blind randomized controlled trial

**DOI:** 10.1186/s13063-022-06328-3

**Published:** 2022-05-07

**Authors:** Yu-guan Zhang, Ying Chen, Yue-Lun Zhang, Jie Yi

**Affiliations:** Department of Anesthesiology, Peking Union Medical College Hospital, Beijing, 100730 China

**Keywords:** Diaphragm ultrasonography, Lung ultrasound, Postoperative pulmonary complications, Postoperative residual curarization

## Abstract

**Background:**

Postoperative residual curarization (PORC) may be a potential risk factor of postoperative pulmonary complications (PPCs), and both of them will lead to adverse consequences on surgical patient recovery. The train-of-four ratio (TOFr) which is detected by acceleromyography of the adductor pollicis is thought as the gold standard for the measurement of PORC. However, diaphragm function recovery may differ from that of the peripheral muscles. Recent studies suggested that diaphragm ultrasonography may be useful to reveal the diaphragm function recovery, and similarly, lung ultrasound was reported for the assessment of PPCs in recent years as well. Sugammadex reversal of neuromuscular blockade is rapid and complete, and there appear to be fewer postoperative complications than with neostigmine. This study aims to compare the effects of neostigmine and sugammadex, on PORC and PPCs employing diaphragm and lung ultrasonography, respectively.

**Methods/design:**

In this prospective, double-blind, randomized controlled trial, patients of the American Society of Anesthesiologists Physical Status I–III, aged over 60, will be enrolled. They will be scheduled to undergo arthroplasty under general anesthesia. All patients will be allocated randomly into two groups, group NEO (neostigmine) and group SUG (sugammadex), using these two drugs for reversing rocuronium. The primary outcome of the study is the incidence of PPCs in the NEO and SUG groups. The secondary outcomes are the evaluation of diaphragm ultrasonography and lung ultrasound, performed by an independent sonographer before anesthesia, and at 10 min and 30 min after extubation in the post-anesthesia care unit, respectively.

**Discussion:**

Elimination of PORC is a priority at the emergence of anesthesia, and it may be associated with reducing postoperative complications like PPCs. Sugammadex was reported to be superior to reverse neuromuscular blockade than neostigmine. Theoretically, complete recovery of neuromuscular function should be indicated by TOFr > 0.9. However, the diaphragm function recovery may not be the same matter, which probably harms pulmonary function. The hypothesis will be proposed that sugammadex is more beneficial than neostigmine to reduce the incidence of PPCs and strongly favorable for the recovery of diaphragm function in our study setting.

**Trial registration:**

ClinicalTrials.gov NCT05040490. Registered on 3 September 2021

**Supplementary Information:**

The online version contains supplementary material available at 10.1186/s13063-022-06328-3.

## Background

Neuromuscular blocking agents (NMBAs) which provide muscle relaxation effects may play an important role in balanced general anesthesia. It could facilitate endotracheal intubation and improve surgical condition. At the end of the surgery, neuromuscular blocking agents are recommended to be reversed completely before tracheal extubation. However, the incidence of postoperative residual curarization (PORC) was reported as high as 57.8% at the time of tracheal extubation and 45.2% in the post-anesthesia care unit (PACU) [1]. Although the use of reversal agents and neuromuscular monitoring has also been recommended, it is conceivable that the situation is far from adequate. Some published surveys documented that only 35% of anesthesiologists thought that quantitative neuromuscular monitoring is necessary [2], and only 17% of them chose to use it [3]. A recent study has shown that nearly 50% of patients did not receive a reversal of neuromuscular block, either neostigmine or sugammadex [4].

Subjective or objective monitors of the neuromuscular block are advocated in clinical practice, of which acceleromyography monitor is the most commonly used to assess neuromuscular block quantitatively in real time. It adopts the train-of-four (TOF) stimulation pattern and displays the TOF ratio (TOFr, T4/T1), which could help the clinician to identify residual muscle blockade by the cutoff value of TOFr < 0.9 [5]. However, the TOF monitoring technique was not routinely carried out in the operating room scenario [6], because these devices require initialization and baseline calibration, and the electrode placement sites sometimes will be interrupted when the patient’s arms are tucked under surgical drapes along the body. Moreover, TOF stimulation may bring unpleasant experiences to patients when they are awake.

Ultrasonography is considered a non-invasive, visualized technique to assist in more and more decision-making for anesthesiologists and intensive care unit (ICU) clinicians. Diaphragm ultrasound has been paid close attention to assess respiratory function, for the diaphragm is the principle respiratory muscle that contributes to more than 60% of tidal volume in every breath [7]. In recent years, several pilot studies have demonstrated that diaphragm ultrasound could be used for the detection of residual muscle dysfunction [8–10]. Our preliminary study also suggested that diaphragmatic excursion (DE) and thickening fraction (TF) during deep breathing have potential efficacy for detecting PORC and were significantly correlated with TOFr in non-thoracic and non-abdominal surgery patients [11]. These results proved that diaphragm ultrasound could provide a feasible measurement to recognize PORC since it is more convenient and comfortable for patients than acceleromyography.

Acetylcholinesterase inhibitors (AChEI) such as neostigmine are commonly administrated to reverse the neuromuscular block effect, which has been considered the gold standard in clinical settings. However, neostigmine has some unavoidable side effects such as bradycardia, cholinergic crisis, and hyperresponsiveness of the bronchus [12]. Moreover, neostigmine may be ineffective for reversing deep neuromuscular block, which may be prone to increase the occurrence of PORC in some circumstances. Some evidence showed that a large dose of neostigmine may lead to transient muscle weakness [13].

Evidence from many retrospective and observational cohort studies have suggested that PORC are associated with many adverse consequences which may contribute to postoperative pulmonary complications (PPCs) [14, 15]. Residual paralysis can cause weakness of the ventilatory muscles, inability to cough, dysfunction of upper airway patency, and impaired swallowing reflex, to increase the risk of hypoxemia, atelectasis, aspiration pneumonia, airway obstruction, and other postoperative pulmonary complications [16, 17]. Therefore, it has been suggested that reducing PORC may decrease the incidence of PPCs. PPCs are harmful and related to poor patient outcomes, especially in high-risk patients, such as elderly patients. The incidence of PPCs was reported to reach 48.3% in elderly patients after administrating neostigmine or without any antagonists [18]. Early and rapid diagnosis is crucial for subsequent treatment. Although thoracic computed tomography (CT) is considered the gold standard of diagnosing PPCs, limitations such as radiation exposure hazards and inconvenient transfer for critical illness patients make it difficult to be routinely performed. Multiple investigations have the evidence that lung ultrasound (LUS) can outperform CT in portability and convenience, and it has been validated as an alternative tool for assisting in detecting atelectasis, pulmonary effusion, and pneumonia, etc. [19, 20].

Sugammadex, the first γ-cyclodextrin drug, is a specific antagonist for neuromuscular block induced by rocuronium. It has been proved by many studies that sugammadex can give a rapid, complete, and long-lasting recovery of muscle strength [21–23]. Compared to neostigmine, sugammadex can minimize the occurrence of PORC and may have the potential to lower the incidence of PPCs [15, 24].

In this prospective double-blinded randomized study, we hypothesize that the incidence of PPCs is lower after reversal with sugammadex than with neostigmine. Considering the incidence of PORC, we postulate that it is lower after reversal with sugammadex than with neostigmine through diaphragm ultrasound. Although the lung ultrasound could not be a substitute for thoracic CT in discerning PPCs, we try to evaluate the difference of changes in lung ultrasound after reversal of neuromuscular block by sugammadex and neostigmine respectively.

## Methods/design

### Study aim

This trial aims to compare the incidence of PORC and PPCs in the sugammadex and neostigmine groups utilizing diaphragm ultrasonography and lung ultrasound and try to conclude whether sugammadex has an advantage over neostigmine in eliminating the occurrence of PORC and PPCs.

### Study design/setting

**Fig. 1:**
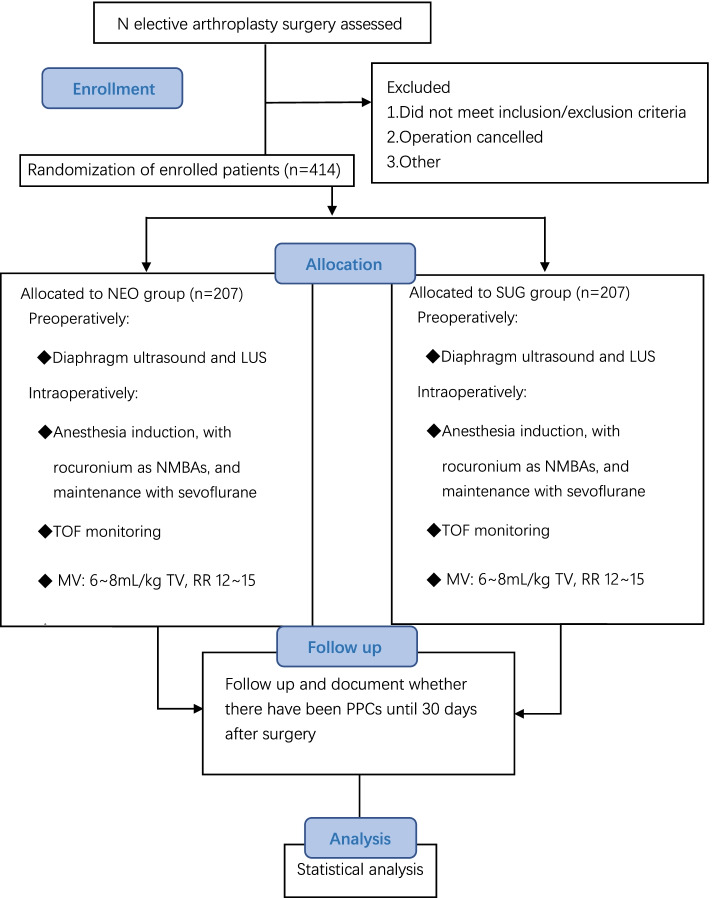
Study flow chart. *t*_1_, preanesthetic period; *t*_2_, induction of anesthesia; *t*_3_, end of surgery; *t*_4_, extubation; *t*_5_, 10 min after extubation; *t*_6_, 30 min after extubation; POD30, postoperative day 30; PPCs, postoperative pulmonary complications

**Fig. 2:**
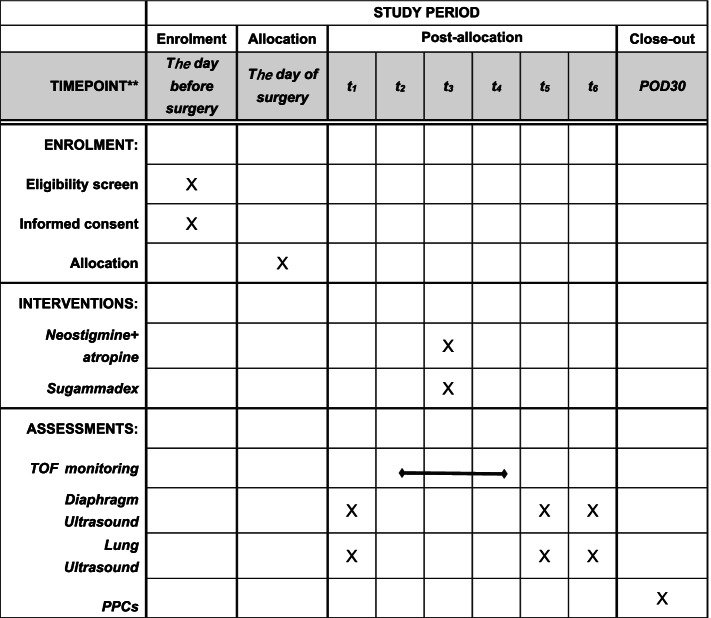
Study timeline. *t*_1_, preanesthetic period; *t*_2_, induction of anesthesia; *t*_3_, end of surgery; *t*_4_, extubation; *t*_5_, 10 min after extubation; *t*_6_, 30 min after extubation; POD30, postoperative day 30; PPCs, postoperative pulmonary complications

This planned study is a prospective, double-blind, randomized controlled trial of superiority, undertaken in Peking Union Medical College Hospital; Fig. 1 is the trial flow chart, and Fig. 2 is the study timeline. The study has been approved by the Ethics Committee of Peking Union Medical College Hospital (No. JS-2674) and has been prospectively registered at ClinicalTrials.gov, NCT05040490, as “Comparison of the Effects of Neostigmine and Sugammadex on Postoperative Residual Curarization and Postoperative Pulmonary Complications Detected by Diaphragm and Lung Ultrasonography” on 3 September 2021. All patients enrolled must sign the patient consent form (see Additional file 1). We used the Standard Protocol Items: Recommendations for International Trials (SPIRIT) reporting guidelines [25], and Additional file 2 is the SPIRIT checklist.

### Participants

Elderly patients are more vulnerable to PPCs, and the incidence of PPCs in patients over 60 years was 19.2% [26]. To facilitate TOF monitoring on the upper limb and enlist relatively older patients, we plan to enroll in total of 414 participants undergoing arthroplasty surgery under general anesthesia, according to the calculation of sample size.

#### Inclusion criteria

The following are the inclusion criteria:

1. American Society of Anesthesiologists (ASA) Physical Status I–III
2. Aged over 60 years
3. Anesthesia induction with rocuronium as NMBAs, maintenance with volatile sevoflurane
4. Scheduled to undertake arthroplasty surgery
5. Signed the informed consent form

#### Exclusion criteria

The following are the exclusion criteria:

1. Those with a history of hepatic or renal disease, chronic or acute alcoholism, allergy or hypersensitivity to sugammadex or neostigmine, current medication with effects on the central nervous system, and a history of dysfunction of the neuromuscular system.
2. Those with diaphragm insufficiency (which can be determined by maximal inspiratory pressures and phrenic nerve stimulation) or massive pleural effusion.
3. Those undergoing upper abdominal laparotomy will be excluded, after which we cannot obtain a satisfactory ultrasound imaging or do not have space for placement of the ultrasonic probe.
4. Those who are extremely old and frail (which can be determined by the FRAIL Scale) and need adjustment of drug dosage.

### Randomization, allocation, and concealment

Once informed consent has been received and the preoperative assessment completed by major anesthetists the day before surgery, patients will be entered into the trial. The randomization procedure will be website-based using random blocks and stratified by the reversal drug the subject takes, which will be done by an independent anesthetist other than major anesthetists. That is, subjects will be randomly allocated to either the sugammadex (SUG) group or the neostigmine (NEO) group in a variable block randomization method, ensuring a 1:1 equal distribution of patients into each group. For allocation concealment, table assignment to the SUG group or the NEO group and drug preparation will be managed by a pharmacist with limited involvement in major anesthesia procedures. Besides, diaphragm ultrasound and LUS will be performed by a skilled sonographer (an independent anesthesiologist) not involved in patient care. Lastly, patients and major anesthetists are both blinded to the treatment assignment.

The randomization codes shall be sealed in duplicate and kept in envelopes by the project leader (PI). Both unblinding is performed by PI who preserved randomization codes. After the data has been blinded and determined to be reliable, the first unblinding will be carried out, only listing the group to which each patient belongs (group A or B). The second unblinding will be performed after the end of the statistical analysis, determining the SUG or NEO group. In this double-blind study, an emergency letter will be attached to every randomization code, illustrating the group and medication of the subject. Emergency letters shall be sealed and kept by PI, and shall not be opened unless there is an emergency and it is necessary to know the medication he/she has received. Once opened, the trial of this patient will be discontinued, and the reason shall be documented in the case report form (CRF).

### Intervention

Patients who meet the enrollment criteria will be randomized 1:1 to either the NEO or the SUG group, receiving NEO or SUG as reversal drugs, respectively, in identical volumes and injectors, with other treatments exactly the same. Patients in the NEO group will receive 50 μg kg^−1^ neostigmine and 15 μg kg^−1^ atropine, while those in the SUG group will receive 2 mg·kg^−1^ sugammadex before the end of operation when TOF count (TOFc) ≥ 2. Drug dosing of anesthesia induction, anesthesia maintenance, and reversal will all be dependent on ideal body weight.

#### TOF monitoring

All enrolled patients will undergo neuromuscular monitoring (adductor of pollicis acceleromyography) with ulnar nerve stimulation using the TOF monitoring device (model No. BeneVision N12; Shenzhen Mindray Biomedical Electronics Co, Ltd., Shenzhen, China). Abduce the forearm of the patient and locate two electrodes along the ulnar nerve, with the distal electrode placed at the intersection of the radial margin of flexor carpi ulnar and proximal wrist stripes, and proximal electrode attached at a distance of 3 cm from the distal one. The transducer should be placed on the ipsilateral distal thumb, perpendicular to the direction of its movement. The device will be calibrated, with T4/T1 at 0.95–1.05, using standard TOF methodology after administration of a hypnotic drug and before muscle relaxation. During the operation, the frequency of TOF stimulation will be 2 Hz, and the wave width will be 200 μs, with a measurement interval of 15 min. During the process, the monitored arm should be free to move. TOFr and TOFc before and after antagonism and immediately before extubation will be recorded on the CRF. No further TOF stimulation will be carried out after extubation. We perform TOF monitoring because TOFr > 0.9 is one of the extubation standards, and we want to verify that diaphragm recovery may be still insufficient despite adequate peripheral TOFr.

#### Induction of anesthesia

After entering the operating room (OR), the WHO surgical checklist will be completed, followed by the establishment of intravenous catheterization, three-lead electrocardiogram, non-invasive blood pressure, pulse oximeter, and other necessary monitoring. General anesthesia will be induced with propofol 2 mg kg^−1^, fentanyl 1.5~2 μg kg^−1^, and rocuronium 0.6 mg kg^−1^ as an intravenous bolus. Tracheal intubation will be performed after TOFc declined to 0.

#### Maintenance of anesthesia

Sevoflurane will be supplied at an age-adjusted end-tidal concentration of 1.0 minimum alveolar concentration (MAC) in an air/oxygen mixture. Fentanyl will be titrated with a bolus of 0.5 μg kg^−1^ every 30 min, to keep an adequate level of analgesia. To maintain neuromuscular block, rocuronium will be re-administered when TOFc exceeds 2 with a bolus of 0.2 mg kg^−1^. Mechanical ventilation will be set at 6~8 ml/kg referred to the ideal body weight as tidal volume and 12~15 rates per minute as respiratory rates according to respiratory carbon dioxide ranging from 30 to approximately 40 mmHg. Extubation will be performed when all the following criteria are met: (1) the patient is awake and can execute simple commands, (2) the patient’s respiratory pattern is regular with a tidal volume of 6–7 ml/kg referred to ideal body weight, and (3) the TOFr is > 0.9.

#### Diaphragm ultrasonography

Before induction of anesthesia, 10 min after extubation, and 30 min after extubation, the right hemidiaphragm of the patient will be evaluated by a specially trained and skilled sonographer (an independent anesthesiologist) using a wisonic ultrasound instrument (model No. Compass; Shenzhen Wisonic Medical Technology Co, Ltd., Shenzhen, China). The sonographer was specifically trained in carrying out diaphragm and lung ultrasonography for 3 months. We only observe and evaluate the right hemidiaphragm because the left one is less accessible and reproducible [27]. The enrolled patient will be placed on the bed in a semi-decubitus position, with the head of the bed raised 45°.

DE of the right hemidiaphragm can be explored by a low-frequency US transducer (convex or phased-array probe) placed along the midclavicular line or below the right costal margin, during respiratory maneuvers such as quiet breathing, voluntary sniffing maneuver, and deep inspiration [28]. DE (amplitude in cm, velocity in cm/s especially during sniffing) can be measured using M-mode ultrasonography. The velocity during the sniffing maneuver can be measured from the baseline to the point of maximum height of inspiration, partially reflecting the potential contractility of the diaphragm [29]. On the zone of apposition (ZOA) to the rib cage for the right hemidiaphragm, it is possible to measure the diaphragmatic thickness (DT) with a high-frequency linear array probe [27]. Diaphragmatic TF can thus be calculated as the ratio, thickness at end-inspiration (TFI) − thickness at end-expiration (TEE) divided by TEE [30]. The diaphragm can be distinguished on the graph by identifying the hyperechoic pleural and peritoneal layers, with these two layers enclosing the hypoechoic diaphragmatic muscle. Three uninterrupted and undisturbed respiratory cycles will be measured and averaged for DE or DT/TF.

#### Lung ultrasound

It is reported that LUS may be used as the primary imaging technique to assess PPCs by distinguishing A lines, B lines, and other typical signs, to assess pulmonary conditions and enhance bedside decision making [31, 32]. In this trial, LUS will be performed on subjects also by Mindray ultrasound instrument (model No. Compass; Shenzhen Wisonic Medical Technology Co, Ltd., Shenzhen, China), before induction of anesthesia, 10 min after extubation, and 30 min after extubation, respectively, by the same anesthesiologist specifically trained in carrying out diaphragm and lung ultrasonography for 3 months.

Each hemithorax can be divided into 3 regions with the anterior and posterior axillary lines—anterior, lateral, and posterior—each of which can be divided into upper and lower parts, that is, 6 examination areas of each hemithorax. The scoring system distinguishes four ventilation patterns as follows: normal aeration (score of 0, presence of lung sliding with A lines and up to two isolated B lines), moderate loss of pulmonary ventilation (score of 1, no fewer than 3 dispersive B lines originated from the pleural cavity), severe loss of pulmonary ventilation (score of 2, multiple coalescing B lines), and pulmonary consolidation (score of 3). As a result, the score of the total lung ranges from 0 to 36. The higher the score, the worse the pulmonary condition, and for each region, the worst visible pattern was recorded.

### Outcomes

#### Primary endpoint

The primary outcome will be the incidence of PPCs 30 days after surgery in neostigmine and sugammadex groups. Using conventional broad criteria for diagnosis of PPCs, any postoperative pulmonary abnormality that adversely affects the clinical course of a patient could be defined as a PPC, including acute respiratory distress syndrome (ARDS), atelectasis, bronchospasm, pleural effusion, pneumothorax, respiratory failure, respiratory infection, and various other forms of upper airway obstruction [33–35] (see Table 1 for more details of its definition). The PI will make a phone call on postoperative day 30 (POD30) to retrospectively check whether there have been PPCs after the surgery till now and make a detailed record of the symptoms, severity, and kind of PPCs on the CRFs.

**Table 1:** Definition of postoperative pulmonary complications (PPCs) [36]

PPCs	Definition
**Acute respiratory distress syndrome (ARDS)**	Acute onset of hypoxemia (partial pressure of oxygen, arterial (PaO_2_)/fraction of inspired oxygen (FiO_2_) ≤ 300 mmHg) with new bilateral infiltrates in the setting of either a normal pulmonary arterial wedge pressure (PAWP ≤ 18 mmHg) or the absence of suspected of left atrial hypertension when PAWP was not available
**Atelectasis**	Collapse of the alveoli; lung opacification with a shift of the mediastinum, hilum, or hemidiaphragm toward the affected area; and compensatory over inflation in the adjacent non-atelectatic lung
**Bronchospasm**	Newly detected expiratory wheeze treated with bronchodilators
**Pleural effusion**	Chest radiograph demonstrating blunting of the costophrenic angle, evidence of displacement of adjacent anatomical structures, or (in supine position) a hazy opacity in one hemithorax with preserved vascular shadows
**Pneumothorax**	A collection of air in the pleural space (the area with no vascular bed surrounding the visceral pleura)
**Respiratory failure**	Postoperative PaO_2_ 60 mmHg on room air, a PaO_2_/FiO_2_ < 300 mmHg, or arterial oxyhemoglobin saturation measured with pulse oximetry < 90% and requiring oxygen therapy
**Respiratory infection**	Treatment with antibiotics for a respiratory infection, plus at least one of the following criteria: new or changed sputum, new or changed lung opacities, fever, and leukocyte count > 12,000/mm^3^

#### Secondary endpoints

The secondary outcome will be the incidence of PORC in the two groups 10 min and 30 min after extubation, using diaphragmatic ultrasound as the method of appraisal. TF during a deep breath of 0.36 was considered as the threshold to exclude diaphragm dysfunction [8, 37–39]. According to our unpublished data, DE during deep breathing of 4 cm or less could also indicate diaphragmatic dysfunction. In this trial, PORC will be defined as TF ≤ 0.36 or DE ≤ 4cm.

#### Other parameters

Sedation scoring of patients (see Table 2 for the Observer’s Assessment of Alertness/Sedation Scale (OAA/S)), duration of hospitalization, year, gender, ASA physical status of patients, dosage of intravenous and inhaling sedative drug/analgesia drug/NMBAs, duration of surgery, and anesthesia.

**Table 2:** The Observer’s Assessment of Alertness/Sedation Scale (OAA/S) [40]

Score	Responsiveness
**5**	Awake and responds to name, spoken in a normal tone
**4**	Lethargic response to name, spoken in a normal tone
**3**	Responds only after the name is called loudly and/or repeatedly
**2**	Responds only after the name is called loudly and after mild shaking of the body
**1**	No response after the name is called loudly with mild shaking

### Statistics

#### Data management and monitoring

Data will be collected using a CRF, and all data from the CRFs will be checked by two investigators before being transferred into an Excel workbook. The digital ultrasound image/video will be copied from the ultrasound machine and stored in a mobile hard disk for review. All data will be kept by the PI independently during and after the trial. An independent Data and Safety Monitoring Committee will oversee the study conduct, report the adverse events, review the quality of collected data, and analyze the fidelity of the study to the protocol on a semiannual basis. The committee consists of a surgeon, an ICU physician, and an anesthesiologist not involved in the study. Also, the Ethics Committee of Peking Union Medical College Hospital will play a role in it. For patients who discontinue or deviate from the protocol, the data of whom will be discarded and that of a new legitimate patient will be recorded for substitution.

#### Sample size calculation

There have been no large-scale studies to confirm the actual incidence of PPCs in patients over 60 years undertaking arthroplasty. Referring to previous literature, the incidence of PPCs is15.4%, 19.2%, and 48.3% in the elderly [18, 26, 41]. To make the results of our study more reliable, we use 15.4% for sample size calculation. The overall incidence is around 5% in all patients undertaking non-cardiac surgery with general anesthesia and endotracheal intubation [15]. The necessary sample size has been calculated using the statistical software PASS 15. The analysis shows that at least 207 patients per group will be necessary (with a 95% confidence interval (CI), a power of 90%, 20% loss of follow-up), that is, 414 patients in total.

#### Statistical analyses

All statistical analyses will be performed by a statistician blinded to the group allocation. Percentages will be calculated for dichotomous data and analyzed by the *χ*^2^ test. Continuous variables will be expressed as mean ± standard deviation and median (ranging from 25th to 75th percentiles) and analyzed with the *T*-test and Mann-Whitney rank sum test. The paired sample *t*-test is used for comparison of the same index. Spearman and Pearson correlation analyses are used for the correlation analysis of normal distribution variables and non-normal distribution variables, respectively. *P* < 0.05 is considered statistically significant. IBM SPSS Statistics 25 is used as our statistical software. Multiple imputation will be used for handling missing data.

## Discussion

From the aspect of pharmacologic profile, the rationale of this comparison study is that PORC may be unavoidable with neostigmine as a reversal agent since it has a relatively short duration and ceiling effect for deep neuromuscular block, as well as some adverse cardiac effects to be concerned. Meanwhile, sugammadex was reported to be superior to reverse aminosteroid neuromuscular blockade, such as rocuronium and vencuronium than traditional anti-cholinesterase, neostigmine.

Logically, the residual effect of neuromuscular block may result in PPCs by mechanisms of ventilatory muscle weakness, impaired swallowing reflex, inability to cough, etc. PPCs are a major contributor to the overall risk of surgery and are associated with high in-hospital mortality. The STRONGER study, which is a retrospective observational matched cohort study, demonstrated that the incidence of PPCs is 30–50% lower by administrating sugammadex than neostigmine [15]. However, another randomized trial that enrolled 200 patients has shown that sugammadex can significantly lower the incidence of PORC, whereas it cannot obtain a significantly decreased rate of PPCs [24]. Such being the case, we do not have conclusive findings. The incidence of PPCs due to multiple perioperative reasons is about 5% in surgical patients [15]; thus, a large sample-sized randomized controlled trial will be necessary to reveal the truth. Our study tried to focus on relative elderly patients who are vulnerable to cardiopulmonary stress and therefore speculated the reduction of PORC by sugammadex will bring better outcomes against PPCs for such an extent population.

Ultrasonography is a novel and useful technique for the evaluation both in diaphragm function and pulmonary complications. Works of literature have suggested that ultrasonography is of good interobserver reproducibility and repeatability if the operator is well trained [29, 42]. DE or DT/TF as endpoints will be measured referring to recent studies of diaphragm ultrasound. We simultaneously perform TOFr monitoring and diaphragm ultrasound in the study for the purpose of verifying that diaphragm recovery may be still insufficient despite adequate peripheral TOFr. Lung ultrasound will be facilitated to detect atelectasis, pneumonia, or pulmonary effusion, which are common pulmonary complications. Unfortunately, although some researches are indicating the high sensibility and specificity of LUS to diagnose atelectasis, pneumothorax, and pleural effusion [31, 32, 43], with a diagnostic accuracy of over 90% [43], there is no consensus on lung ultrasound to diagnose PPCs yet. We, therefore, aim to link the incidence of PPCs to lung ultrasound by analyzing the postoperative ultrasound imaging score and follow-up procedures straight into 30 days after their surgeries.

In summary, this randomized double-blind controlled trial may be the first study that provides important information on the comparison of incidence of PORC and PPCs between neostigmine and sugammadex through two novel and useful approaches, diaphragm ultrasonography, and lung ultrasound.

## Trial status

The date of first enrollment was August 31, 2021. Participants are currently being recruited and enrolled. The approximate date when recruitment will be complete is September 30, 2022.

## Supplementary Information

**Additional file 1.** Patient consent form (in Chinese).

**Additional file 2.** SPIRIT checklist: Recommended items to address in a clinical trial protocol.

## Data Availability

All the compiled CRFs will be archived. After this study is complete, the final trial dataset and statistical codes will be available from the corresponding authors upon reasonable request, except for participants’ personal information.

## Abbreviations

AChEI: Acetylcholinesterase inhibitors
ARDS: Acute respiratory distress syndrome
ASA: American Society of Anesthesiologists
CI: Confidence interval
CRF: Case report form
CT: Computed tomography
DE: Diaphragmatic excursion
DT: Diaphragmatic thickness
FiO_2_: Fraction of inspired oxygen
ICU: Intensive care unit
LUS: Lung ultrasound
MAC: Minimum alveolar concentration
NEO: Neostigmine
NMBAs: Neuromuscular blocking agents
OAA/S: Observer’s Assessment of Alertness/Sedation Scale
PACU: Post-anesthesia care unit
PaO_2_: Arterial partial pressure of oxygen
PAWP: Pulmonary arterial wedge pressure
PI: Project leader
POD30: Postoperative day 30
PORC: Postoperative residual curarization
PPCs: Postoperative pulmonary complications
SUG: Sugammadex
TF: Thickening fraction
TEE: Thickness at end expiration
TFI: Thickness at end inspiration
TOF: Train-of-four
TOFc: Train-of-four count
TOFr: Train-of-four ratio
ZOA: Zone of apposition
