# Supplementary material for: Comparison of the effects of neostigmine and sugammadex on postoperative residual curarization and postoperative pulmonary complications by means of diaphragm and lung ultrasonography: a study protocol for prospective double-blind randomized controlled trial
Source: Trials. 2022 May 7;23:376. doi: 10.1186/s13063-022-06328-3 (PMC9077960; doi:10.1186/s13063-022-06328-3)
Supplement: Supplementary file 1 — Additional file 1. Patient consent form (in Chinese). [file 13063_2022_6328_MOESM1_ESM.docx]

**知情同意书版本2.0**

膈肌超声对舒更葡糖钠或新斯的明拮抗后肌松残余的监测及肺部并发症发生率的随机双盲对照研究

知情同意书

亲爱的患者：

您们好，您因为原发病于我院在全身麻醉气管插管术下行手术治疗。我们将诚挚邀请您参加一项临床研究：膈肌超声对舒更葡糖钠或新斯的明拮抗后肌松残余的监测及肺部并发症发生率的随机双盲对照研究。本研究方案已经得到中国医学科学院北京协和医院医学伦理委员会审核，同意进行该项临床研究。

在您决定是否参加这项研究之前，请尽量仔细阅读以下内容。它可以帮助您了解该项研究以及为何要进行这项研究，研究的程序和期限，参加研究后可能给您带来的益处、风险和不适。如果您愿意也可以和您的家属、朋友一起讨论，或者请医生给予解释，帮助您做出决定。

一、**研究背景和研究目的**

既往研究报道全麻术后肌松残余的发生率较高，2016年我国的RECITE研究发现拔管后肌松残余可达57.8%，在恢复室肌松残余的发生率也高达45%以上。严重的肌松残余与患者术后肺部并发症（post-operative pulmonary complications ，POPC）密切相关。新斯的明作为胆碱酯酶抑制剂是目前最常用肌松拮抗药，但其在临床使用也有其局限性。舒更葡糖钠是新型的特异性肌松拮抗剂，大量的研究已经报道舒更葡糖钠有更为快速、彻底逆转肌松，恢复自主呼吸的特点。舒更葡糖钠也能够快速逆转深肌松，改善腹腔镜手术预后。膈肌超声和肺部超声作为无创的监测方式，可以对膈肌和肺部情况进行评估。

本研究的目标旨通过随机对照双盲研究完成：膈肌超声和经典肌松监测对全身麻醉、罗库溴铵诱导的患者，舒更葡糖钠拮抗和新斯的明拮抗后肌松残余的评估；肺部超声评估全身麻醉罗库溴铵诱导的患者，舒更葡糖钠拮抗和新斯的明拮抗后肺部并发症的情况；两组拮抗药物应用后，肌松残余组和肌松非残余组中肺部并发症的发生率。

二、**哪些人不宜参加研究**

本研究有严格的纳入标准和排除标准，凡不符合纳入标准的患者不宜参加本研究，另外还有1）正在参加其它临床研究的患者；2）研究人员认为其他原因不适合临床研究者。

**三、如果参加研究将需要做什么？**

1. 在您入选研究前，医生将询问、记录您的病史。您是合格的纳入者，自愿参与该研究，签署知情同意书。

2. 若您自愿参加研究，我们根据指定的随机表，将您纳入两个研究组的其中一组，接受两种不同拮抗药之一的术后常规拮抗。任何一组的术后拮抗药物均为现临床常规拮抗药物。

3. 若您自愿参加研究，我们进行术后随访以了解治疗的治疗效果、我们会详细安排您的治疗流程。

**四、参加研究可能的受益**

如果您参加了该研究，在您术后恢复及治疗中，将获得以下支持。包括：

1. 更加完善的诊疗措施：包括更加细致和完善的术后访视及相关治疗。对于围术期麻醉相关的问题进行详尽的解答及治疗。围术期麻醉相关并发症更完善的监测，及时的发现和处理对术后早期康复有更大的帮助。

2. 专门的随访与咨询：本项目将设立随访，让您得到及时、全面的长达术后1个月的病情咨询与监测，并对您在术后恢复中相关问题进行及时的回答和处理。

**五、参加研究可能的风险、不良反应和不适、不方便**

因本研究手术方式及麻醉方式为目前临床常规开展的项目，因此参加本研究本身不会额外增加患者风险。对于舒更葡糖钠或新斯的明两个治疗组中≥10%患者发生最常见药物相关临床不良事件分别为切口部位疼痛（23%和23%）、头晕（9%和19%）、发热（13%和14%）以及恶心（8%和12%）。舒更葡糖钠的不良反应比临床常规应用的新斯的明的发生率要有所降低，不增加其他风险。

临床治疗后，包括在研究期间如果患者出现任何不适，或者病情出现新的变化，或任何意外情况，不管是否与研究有关，均应及时通知您的医生，医生将对此做出判断并给予适当的医疗处理。一旦发生由药物临床试验导致的损害时可以获得免费医疗或获得其应得的伤害赔偿。

**六、有关费用**

由于本研究所涉及的麻醉方法是日常临床全身麻醉应用的常规方法，并未额外增加手术风险和治疗费用，因此，所有治疗费用均由患者自行负担。舒更葡糖钠现阶段为自费药品，需要患者自行负担药物相关费用。对于患者同时合并的其他疾病所需要的治疗和检查，也将不在免费范围之内。

**七、个人信息是保密的吗？**

您的医疗记录（研究病历/CRF、化验单等）将完整地保存在您所就诊的医院。医生会将化验及其他检查结果记录在您的病历上。研究者、伦理委员会和药品监督管理部门将被允许查阅您的医疗记录。记录您所有的个人信息，包括姓名、电话、电子邮件、住址等均不会出现在电子数据库中，任何有关本研究结果的公开报告将不会披露您的个人身份及信息。我们将在法律允许的范围内，尽一切努力保护您的个人医疗资料的隐私。

**八、怎样获得更多的信息？**

您可以在任何时间提出有关本研究的任何问题，并得到相应的解答。咨询电话（研究者电话）：18810262025；且您有权就有关您的权利或相关风险等问题进行咨询，咨询电话（伦理审查委员会电话）：69154494.如果在研究过程中任何重要的新信息，可能影响您继续参加研究的意愿时，您的医生将会及时通知您。

**九、可以自愿选择参加研究和中途退出研究**

是否参加研究完全取决于您的意愿。您可以拒绝参加此项研究，或在研究过程中的任何时间退出本研究，这都不会影响您和医生间的关系，都不会影响对您的医疗或有其他方面利益的损失。

出于对您的最大利益考虑，医生或研究者可能在研究过程中随时中止您继续参加本研究。

**十、现在该做什么？**

是否参加本项研究由您（和您家人）决定。

在您做出参加研究决定前，请尽可能向您的医生询问有关问题。

感谢您阅读以上材料。如果您决定参加本项目，请告诉您的医生，他/她会为您安排一切有关研究的事务。请您保留这份资料。

同意声明

我已经阅读了上述有关本研究的介绍，并且有机会就此项研究与医生讨论并提出有关问题。我提出的所有问题都得到了满意的答复。

我知道参加本研究可能产生的风险和受益。我知晓参加本研究是自愿的，我确认已有充足的时间对此进行考虑，而且明白：

我可以随时向医生咨询更多的信息。

我可以随时退出本研究，而不会受到歧视或报复，医疗待遇与权益不会受到任何影响。

我同样清楚，如果我中途退出研究，特别是由于药物的原因使我退出研究时，我若将我的病情变化告诉医生，完成相应的体格检查和理化检查，这将对整个研究十分有利。

如果因病情变化我需要采取任何其他药物治疗，我会在事先征求医生的意见，或在事后如实告诉医生。

我同意药品监督管理部门、伦理委员会或申办者代表查阅我的研究资料。

我将获得一份经过签名并注明日期的知情同意书副本。

最后，我决定同意参加本项研究，并保证尽量遵从医嘱。

患者签字： 日期： 年 月 日

或

受试者家属签字： 与受试者关系： 日期： 年 月 日

我确定已向患者解释了本研究的详细情况，包括其权力以及可能的受益和风险，并给其一份签署过的知情同意书副本。

研究者签字： 日期： 年 月 日
